# How Do the Children Play? The Influence of Playground Type on Children’s Play Styles

**DOI:** 10.3389/fpsyg.2021.703940

**Published:** 2021-10-13

**Authors:** Tina L. Stanton-Chapman, Eric L. Schmidt

**Affiliations:** ^1^Early Childhood and Human Development School of Education, Criminal Justice, and Human Services, University of Cincinnati, Cincinnati, OH, United States; ^2^Playground Equipment Services, Cincinnati, OH, United States

**Keywords:** play, playgrounds, environmental context, peer relationships, play skills, setting

## Abstract

Using a mixed-method design, the aims of the current study were to develop an in-depth understanding of (1) children’s social play behaviors on school and community playgrounds, (2) the duration with which children play within varying social play categories, and (3) assessing children’s perspectives of playground activities, their peer relationships, and recommendations for new playgrounds. Six participants were observed for five 30-min observations on a school playground and for five 30-min observations on a community playground. Participants were also interviewed about their experiences and preferences on school and community playgrounds. The direct observation results support and extend previous work, indicating that children’s play skill competence varies by setting. Children demonstrated higher levels of associative and cooperative play on the school playground, but higher levels of solitary and parallel play on the community playground. This difference in play styles by playground appears to be a function of available play partners and is explained by the interview data, which found that children are not comfortable playing with children they do not know.

## Introduction

The developmental importance of play is well-documented (e.g., Johnson et al., 1999; Holst, 2017; Stone, 2017). Over the years, many researchers have attempted to define *play* and these definitions vary widely. Most researchers agree that play encompasses a combination of characteristics, rather than the presence or absence of one defining characteristic (Stone, 2017). Fromberg and Bergen (2006) offered the following characteristics of play—symbolic, meaningful, active, pleasurable, voluntary, intrinsically motivated, rule-governed, and episodic. Eberle (2014), when defining play, included six elements, which characterize play—anticipation, surprise, pleasure, understanding, strength, and poise, while Rubin et al. (1983) characterized play as intrinsically motivated, controlled by the players, concerned with the process rather than the product, non-literal, free of externally imposed rules, and characterized by the active engagement of players. Regardless of the definition, it is clear that play allows children to learn skills that they previously have not experienced, master newly acquired skills, and adapt the skills that have been learned and mastered and applies them to new situations (Malone, 1999; Holt et al., 2015).

### Physical Environment and Affordances

The physical environment is believed to be a critical factor in children’s play and development. Indeed, play settings have been found to positively influence children’s well-being and physical activity levels (Sando, 2019). The correlation between play settings and children’s well-being and physical activity levels is best described using Gibson’s (1950) theoretical framework known as the *Theory of Affordance*. The affordances of the physical environment are what the environment offers the child and the complementarity of the child and the environment. For example, a preschool classroom’s affordances are the physical size of the classroom, the toys and materials available, the number of peers for potential play partners, and adults who set up the play environment, ensure its overall safety, manage the day-to-day operations, and interact with the students.

Children play in a number of settings including the home, classroom, playground, and local neighborhood. Each of these settings offers its own affordances, which impact children’s well-being and physical activity levels in different ways. Of particular interest, in the current study, is the outdoor playground as a physical play environment. Playgrounds offer several affordances and play behaviors to children—climbing-on, climbing-off, jumping-on, jumping-off, swinging-on, swinging-off, running-on, and running-off (Sandseter, 2009). A child’s ability to move freely around the playground environment without an adult’s control not only allows a child to actualize a playground’s affordances but also increases his or her physical activity levels (Graham et al., 2021).

### Playground Types and Social Play

In the USA, there are two types of playgrounds—a *school playground* and a *community playground*. A *school playground* is an outdoor play environment located within the property lines of an elementary school. Its purpose is to provide a place for elementary students (ages 5years to 11years) to play outdoors during recess time. Community playgrounds fall under two categories—a *neighborhood* and a *destination* playground. A *neighborhood* playground is defined as a playground that is built in a residential community (Brown and Burger, 1984). Its purpose is to provide children with a place to play within walking distance of where they live. A *destination* playground is defined as a playground that is built in a place where playground patrons are expected to drive or take public transportation to utilize the play space (Brown and Burger, 1984). The play area and equipment may be the same or vary at neighborhood and destination playgrounds (Rimmer, 2005).

Previous studies examining school and community playgrounds have focused on the impact of playground spatial features on their overall play value (e.g., Czalczynska-Podolska, 2014), how they meet the needs of children with disabilities (e.g., Yuill et al., 2007; Stanton-Chapman and Schmidt, 2016, 2017), children’s playground equipment preference (e.g., Bourke and Sargisson, 2014), the influence of playgrounds and playground equipment on children’s physical activity levels, childhood obesity rates, or well-being (e.g., Sallis et al., 2001; Ridgers et al., 2006; Farley et al., 2008; Uys et al., 2015; Sando, 2019), and playgrounds that offer opportunities for risk and their appeal to children (Lasenby-Lessard and Morrongiello, 2011; Lasenby-Lessard et al., 2013). While playground settings have become a field of growing interest in research on child outcomes, there are a lack of studies assessing how the type of playground setting (school playground; community playground) influences the differential expression on children’s play behaviors.

Although play behavior is most often classified by researchers cognitively with terms such as *functional play, dramatic play*, or *constructive play*, play that takes place on playground settings is best categorized by social definitions given its dependency on social interactions with peers (Wilson, 2015). Parten’s stages of play (e.g., *parallel play, associative play, onlooker play;* Parten, 1932) are still considered the ideal when describing preschool children’s social play behavior during free play on contexts such as school and community playgrounds (Jarusriboonchai et al., 2019). Contextual factors such as the availability of loose equipment or teacher supervision are known to impact children’s play behaviors on a given playground--school OR community (Wilson, 2015). The influence and overall impact of contextual factors on children’s cognitive and social play behaviors have also been studied in laboratory playrooms, classrooms, and home settings (e.g., Guralnick and Groom, 1988; Malone and Stoneman, 1990; Malone et al., 1994; Fewell et al., 1997; Malone and Langone, 1998; Malone, 2006). However, little is known about children’s play behaviors across two different playground settings at this time. The comparison of school and community playgrounds on children’s play can provide additional insight into how children’s developmental abilities are influenced by contextual conditions. Using a mixed-methods design, the aims of the current study were to develop an in-depth understanding of (1) children’s social play behaviors on school and community playgrounds, (2) the duration with which children play within varying social play categories, and (3) assessing children’s perspectives of playground activities, their peer relationships, and recommendations for new playgrounds.

## Materials and Methods

The present investigation took place at a school playground and a community playground in a large midwestern city in the USA. The six children (three boys and three girls) who participated in the study were enrolled at the elementary school where the school playground was located. The elementary school serviced children in prekindergarten (PK) through fifth grade. Participants in the current study were in prekindergarten (classrooms for 4-year-olds) and lived within 15min of the community playground. The characteristics of each child are provided in Table 1. The names of the children are pseudonyms. None of the children had identified disabilities including attention deficit hyperactivity disorder.

**Table 1:** Demographic information on study participants (*N*=6).

Participants	Gender	Age at start of study (years/months)	Grade	Race/ethnicity
Deiondre	Male	4.7	PK	African-American
Julia	Female	5.1	PK	Latina
Tamisha	Female	4.4	PK	African-American
Tobias	Male	5.2	PK	Bi-racial
Flora	Female	4.0	PK	Caucasian
Beau	Male	4.5	PK	Caucasian

A purposive sampling strategy covering participants representing different demographical backgrounds was implemented (Palinkas et al., 2016). To conduct the purposive sampling, the authors followed a series of steps. First, the authors selected the community playground. The community playground was a convenience purposive sample as the playground previously served as a study site for the authors, and the local community government’s ethical board provided approval for additional studies to take place at the playground. Second, the authors searched for elementary schools with PK programs within 15-min driving distance of the playground. Twelve elementary schools met the criteria. Third, to reflect the diversity of culture and conditions, the authors targeted elementary schools with more equivalent rates of race and ethnicity and a free or reduced lunch rate of 50.0% or above. Two elementary schools met the criteria. Fourth, the first author approached the principals of both elementary schools for permission to participate in the study. Only one principal agreed. Fifth, the first author met with the two PK teachers who taught at the elementary school whose principal agreed to participate in the study. The first author discussed the purpose of the study and asked for their permission to send consent forms home to the 30 prekindergarten students enrolled in the elementary school (2 PK classrooms; 15 students per classroom). Both teachers agreed to send consent forms home to their students. Last, the first three boys and the first three girls whose caregivers provided consent for their children to participate in the study *and* agreed to transport their child to the community playground at mutually agreed-upon times were selected for participation. The enrollment of six participants sufficiently adheres to mixed-methods research design rigor (Creswell and Clark, 2017). The first three boys and the first three girls selected for participation did not include individuals with disabilities. Since the current study did not have a disability focus or purpose, this participation pool was appropriate.

The elementary school, where the participants were enrolled, is diverse and has a 53.6% free or reduced lunch rate. According to state test scores, 66% of enrolled students are proficient in reading and math. Participants did not play on the school or community playground with another participant in the study or a sibling. To reduce possible behavioral issues between siblings or peers, caregivers agreed to not to bring another child with them to the community playground. Caregivers and children were not compensated for their time in the study.

### Setting

**Figure 1:**
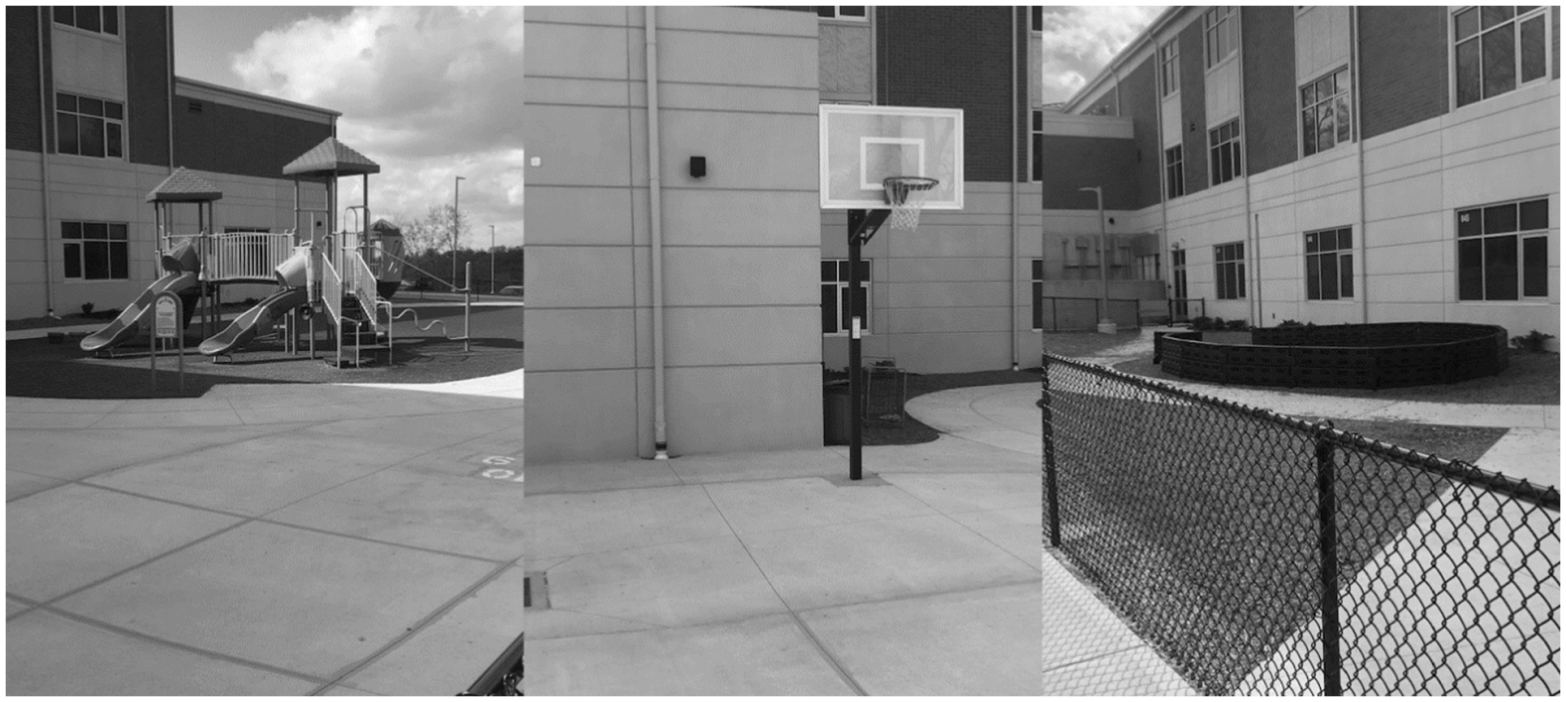
Visual of the school playground.

**Figure 2:**
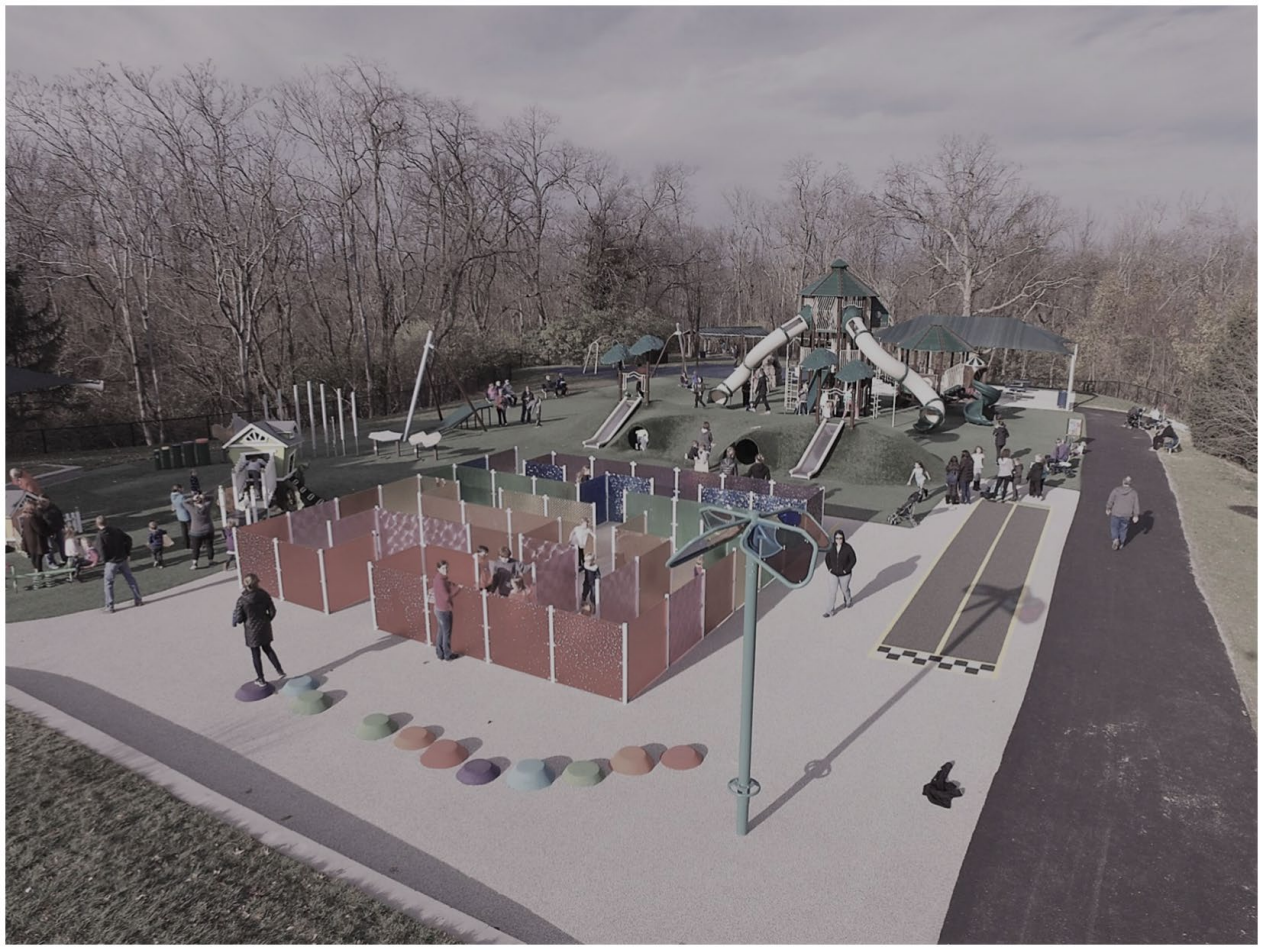
Visual of the community playground.

Figures 1, 2 provide visuals of the school and community playgrounds.

#### School Playground

The school playground was a long, irregular rectangle area of 6,426 sq. ft^2^ connected by a concrete walkway. It contained three observed play areas: (1) a *turf and concrete area* consisting of an installed playground structure surrounded by artificial turf and a concrete path for walking and tricycles (never observed during the current study); (2) an *open equipped concrete area* with two unshaded basketball hoops, two painted four-square games, and two painted hopscotch areas; and (3) a *mulch-covered area* consisting of a circular gaga ball pit. The installed playground structure was a connected network of two slides, a walking bridge, two platforms, and a climbing bar. It did not contain a tunnel or swings. The basketball hoops were lower, and the areas surrounding them were smaller than regulation size—as appropriate for preschool children. The gaga ball pit was age-appropriate in size and allowed students to enter and exit quickly and safely. The school provided basketballs and gaga balls daily, and they were available in the areas where they were to be used. The two PK classrooms shared similar recess times, and 30 students including the study’s participants were on the school playground at the same time.

#### Community Playground

The community playground is best described as a destination, universal playground with a focus on the abilities of *every* child across the age span. The playground also provided ample sensory activities for children who have sensory needs. The overall area of the playground is 16,000 sq. ft^2^. Sixty parking spaces are available for parking, and all 60 are usually full on any given day. It is an extremely busy playground. The playground has eight distinct play zones: (1) an *infant/toddler play area* with playhouses; (2) a *musical instrument area*; (3) a *sensory maze* which provides tactile and visual stimulation; (4) a *turf hill with metal slides and tunnels*; (5) a *14-foot mega-tower with slides*; (6) a *zipline*; (7) a *traditional swing area with a toddler swing and an accessible swing*; and (8) a *disk swing* which accommodates multiple children. Seating areas and a shade structure are provided for family use. Over 85% of the playground is ground level, and the entire playground facility is surfaced using pour-in-place surfacing or artificial turf. Both surfacing types allow mobility devices to move freely throughout the playground.

### Research Design

A descriptive case design (mixed-methods) was used, and quantitative and qualitative data were collected and analyzed (Creswell, 2009; Creswell and Clark, 2017). Descriptive case studies describe a population, situation, or phenomenon that is being studied to learn the *how, what, when*, and *where* (Dyson, 1985). For this investigation, a descriptive-comparative case design was used to investigate two variables (school playground; community playground) to better understand how each playground environment promotes children’s social play. Six children were participated in the current study to offer a “redundancy of cases” to build a stronger understanding and a more compelling argument for the significance of the research (Barone, 2004).

### Procedures and Data Analysis

#### Direct Observation

A total of 60 individual field observations were conducted: 30 observations took place on the school playground and 30 observations were recorded on the community playground. An equal number of observations per child and setting were completed. Each child was observed five times on the school playground and five times on the community playground. Each observation lasted 30min. This procedure yielded an observation time of 150min per playground per child (300 total observational minutes per child). The period of observation lasted from March 18, 2019, to May 24, 2019, always from 11:00AM until 12:00PM. This time frame accounted for the assigned recess times at the elementary school. School playground observations took place on school days for the school playground and on weekends for the community playground.

Two observers (one primary, one reliability) served as coders. Both coders were undergraduate early childhood education majors with experience in observing young children as they played. Both observers volunteered in the participants’ classrooms and visited both playgrounds prior to the study in order to become acquainted with participants and settings. Participants and their peers were told: (1) the observers could not participate in their play and (2) the observers may follow them around the playground. Participants quickly habituated to the situation and the “following” observer.

For the purpose of the current study, Parten’s (1932) Play Categories were used to characterize the participants’ play. Table 2 provides the categories of play and their respective examples. A duration recording conducted on an iPad was used to code the play categories. Duration recording involves collecting data on how long a behavior occurs (Ayres and Gast, 2010). A *duration per occurrence* tactic was employed—the coders started and stopped the stopwatch (on the iPad) with each occurrence of the play category (e.g., solitary play, associative play, onlooker play). Coders were trained to use these play categories, the iPad, and the iPad’s stopwatch through video and live observations and discussed disagreements as they occurred. At the conclusion of the training, the reliability criterion required coders to achieve 80% or above reliability on both video and live observation. Training coders took approximately 8h.

**Table 2:** Categories of play and their respective examples.

Categories of Play	Definition	Examples on the playground setting
Unoccupied Play (UP)	The child is not playing with an object or with other children The child might be standing, wandering, or sitting	The child is walking the perimeter of the playground The child is sitting on the side of the playground area The child picks mulch or grass
Solitary Play (SP)	The child is playing alone He/she is using playground equipment but is not near or playing with other children	The child is shooting baskets by himself/herself The child is playing on the playground structure by himself/herself The child is using the zipline
Onlooker Play (OP)	The child is watching other children play but makes no attempt to join in The child may make comments to the children who are playing but continues to watch their play	The child is watching other children play on the turf hill or using the zipline The child is watching other children play in the gaga ball pit
Parallel Play (PP)	The child is playing next to another child who is using the same play equipment or materials The child does not interact with the other child and plays with the play equipment as he/she wants	The child is swinging on the swings The child is using the same items as another child on the playground structure, but they are not interacting with one another
Associative Play (AP)	The child is playing with another child or children They interact and play with the same equipment and materials Play is loosely organized. Children may assign roles but may not follow these roles as these roles as they play	Two or more children using the playground structure and one child asks another child do the same thing as him/her (e.g., go down the slide like me). The play is brief Two or more children are chasing one another on the playground. The play is brief
Cooperative Play (CP)	The child is playing with other children They interact and play with the same equipment or materials. Games are common Play is very organized. Children may assign roles and will follow these roles as they play	A child playing in the gaga ball pit with other children A child taking turns shooting baskets with another child Two children riding on the disk swing

At the conclusion of the training, the coders were randomly assigned three participants. The coder served as the primary coder for these three participants and served as the reliability coder for the remaining three participants. For example, if Coder A served as the primary coder for Deiondre, Julia, and Tamisha, then Coder A served as the reliability coder for Tobias, Flora, and Beau. Twenty percent (*N*=34) of all total observations were double-coded for reliability purposes. The direct observation protocol was as follows: (1) turn on iPad, enter child’s name on the observational form, and enter school or community playground on the observational form, (2) locate target child on the playground and proceed to child’s approximate location, (3) turn on stopwatch, (4) code the play category of the child using the appropriate initials (refer to Table 2), (5) when the child’s play behavior changes to a new play category, the coder stops the stopwatch and then restarts the stopwatch to input a new play category. Step 5 is repeated each time the child changes his/her play behavior to a new play category until the 30-min observation is complete.

#### Interviews

Individual interviews with the children were conducted in person by the first author at the community playground. Visits to each child’s preschool classroom were made prior to the interviews. This provided the opportunity to meet the children and interact with them during play-based activities. As noted by Lambert et al. (2013), meeting children prior to an interview increases familiarity and begins the process of rapport.

In all cases, interviews took place at a picnic table at the community playground. Children played on the community playground for 30min before the interview began. Using age-appropriate language, the interviewer started by asking if the children were willing to answer a few questions about the playground they just played on and about their school playground. All six children agreed to participate in the interview portion of the study. Semi-structured interviews were conducted using a series of open-ended questions, probes, and opportunities for the children to share their opinions and experiences on playground play.

Based on the principles of qualitative interviewing, interview questions remained open to revision through the course of data collection (Eder and Corsaro, 1999). Consequently, the interviews maintained an informal quality, providing children with flexibility to discuss a variety of topics. The informal structure of qualitative interviewing allows the researcher to “*gain access to the knowledge, experience, and perspectives of research subjects, rather than organizing the beliefs, experiences, and perspectives of research subjects into preset categories like quantitative interviewing does,”* (Kelly, 2013, p. 309). The total amount of time spent with each child was 15min. A timer was used to end the interview at 15min. The interview questions explored four topic areas and were not always presented in a specific sequence. The four topic areas included: (1) participant’s favorite activities at each playground (*What do you like to play on at the school and name of community playground?)*; (2) participant’s least favorite activities at each playground (*What do you not like to play on at the school and name of community playground?)*; (3) a description of their peer relationships (*who do you play with on the playground?)*; and (4) their recommendations for playground builders (*what changes would you make to your school’s playground?”* and “*what changes would you make to the* *name of community playground*?). As typical in qualitative interviewing, the interview protocol was semi-structured using the four topic areas as a guide but allowing the research participants to guide the overall discussion (Kelly, 2013). The first author, as interviewer, adhered to the following qualitative research design interviewing protocol standards: (1) be an active listener; (2) ask probing questions as a means to clarify participant answers and explore the information in-depth (e.g., “Can you tell me more about that?”); (3) ask follow-up questions if information is missing (e.g., child did not discuss one or more of the four topics on their own accord) or more elaboration is needed; (4) avoid inserting yourself or your opinion into the conversation; and (5) allow the child to lead the interview (e.g., if the child branched into one or more of the four topics on their own without being questioned directly, the interviewer allowed the child to continue on with the topic). For example, if a child discussed her favorite activities at the school playground and then immediately discussed her least favorite activities, she was not interrupted. She was allowed to continue the discussion.

Each interview was audio-recorded and transcribed verbatim. Creswell’s (2009) transcription analysis procedure was applied: (1) obtaining a general sense of the data to a more detailed explanation; (2) codes represented inductive concepts derived directly from the interview topics; (3) ongoing data-checking; and (4) revision of interview questions was conducted based on emerging themes. Inter-rater reliability was established by having two graduate students review emerging themes four times after every three interviews. Revisions to the interview schedule were made based on queries or emerging evidence. For example, interview questions were revised to include probing of least favorite activities at each playground. Supplementary questioning in this area afforded the opportunity to explore the origins of where children played or did not play on each playground.

Coding categories were continually refined and developed until a thorough interpretation of the data was reached. Key themes were identified through consensus and mutual agreement between coders. No discrepancies in coding appeared at any point during coding. Qualitative rigor was achieved through participants’ direct quotes supporting the analysis, peer debriefing, and negative case analysis. Preliminary findings were reviewed by play experts (PhD researchers who have studied children’s play throughout their careers). These discussions provided additional validation of children’s playground play.

A graduate student with experience in qualitative analysis used NVivo 8 (QSR International, 2008) to facilitate the coding process. Interview data were subjected to analyses including theme generation. These analyses addressed similarities and differences between the children including conflicting information.

## Results

### Direct Observation Data

#### IOA and Cohen’s Kappa

Interobserver agreement (IOA) and Cohen’s Kappa (Cohen, 1960) were calculated on the overall percent of agreement between the two undergraduate coders who observed participants playing on the school and community playgrounds using Parten’s play categories. Mean IOA for Deiondre was 88% (range=70 to 100%); mean IOA for Julia was 93% (range=80 to 90%). Mean IOA for Tamisha was 89% (range=82 to 100%). Mean IOA for Tobias was 82% (range=68 to 100%). Mean IOA for Flora was 88% (range=78 to 100%). Mean IOA for Beau was 93% (range=80 to 100%). Mean IOA did not vary by school or community playground.

Cohen’s Kappa values range from +1 to −1, with high positive values indicating higher agreement by observers than expected by chance and high negative values indicating evidence of observers agreeing less frequently than expected by chance (Cohen, 1960). Kappa values are interpreted in the following manner: 0.21 to 0.40 is fair agreement; 0.41 to 0.60 is moderate agreement; 0.71 to 0.80 is substantial agreement; and 0.81 to 1.00 is almost perfect agreement. In the current study, Kappa agreement was 0.88 for Deiondre (almost perfect); 0.97 for Julia (almost perfect); 0.93 for Tamisha (almost perfect); 0.75 for Tobias (substantial); 0.90 for Flora (almost perfect); and 0.92 for Beau (almost perfect).

#### Parten’s Play Categories

Table 3 shows the duration of time that each child spent in each play category by type of playground. These data show higher levels of associative and cooperative play on the school playground. Higher levels of solitary and parallel play occurred on the community playground. Four children (Deiondre—74%, Julia—89%, Tamisha—100%, Tobias—94%) played cooperatively with peers for the majority of time when they visited the school playground. Beau played cooperatively (53%) and associatively (43%) when he visited the school playground. Flora was more likely to demonstrate associative style play (57%) while on the school playground.

**Table 3:** Duration of time that each participant spent in each play category by type of playground.

Name	Category of play	Type of playground									
		School observations (in minutes)					Community playground observations (in minutes)				
		1	2	3	4	5	1	2	3	4	5
**Deiondre**											
	Unoccupied	0	0	0	0	0	0	0	0	0	0
	Solitary	0	0	0	2.0	0	18.0	17.0	22.0	25.0	16.0
	Onlooker	1.0	0	0	1.0	0	6.0	10.0	0	2.0	2.0
	Parallel	0	5.0	1.0	0	0	6.0	3.0	8.0	3.0	9.0
	Associative	4.0	7.0	2.0	10.0	6.0	0	0	0	0	3.0
	Cooperative	25.0	18.0	27.0	17.0	24.0	0	0	0	0	0
**Julia**											
	Unoccupied	0	0	0	0	0	0	0	1.0	0	0
	Solitary	2.0	0	5.0	0	0	12.0	23.0	25.0	19.0	16.0
	Onlooker	0	0	0	0	0	2.0	0	1.0	0	0
	Parallel	2.0	0	0	0	0	15.0	7.0	4.0	10.0	6.0
	Associative	3.0	1.0	3.0	0	0	1.0	0	0	0	2.0
	Cooperative	23.0	29.0	22.0	30.0	30.0	0	0	0	1.0	6.0
Tamisha											
	Unoccupied	0	0	0	0	0	0	0	0	0	0
	Solitary	0	0	0	0	0	20.0	17.0	24.0	14.0	27.0
	Onlooker	0	0	0	0	0	0	1.0	3.0	2.0	0
	Parallel	0	0	0	0	0	6.0	10.0	3.0	14.0	3.0
	Associative	0	0	0	0	0	4.0	2.0	0	0	0
	Cooperative	30.0	30.0	30.0	30.0	30.0	0	0	0	0	0
**Tobias**											
	Unoccupied	0	0	0	0	0	0	0	0	0	0
	Solitary	0	0	4.0	0	0	16.0	11.0	23.0	19.0	28.0
	Onlooker	0	0	0	0	0	12.0	2.0	0	0	0
	Parallel	0	0	0	0	0	1.0	8.0	5.0	4.0	2.0
	Associative	0	3.0	0	0	0	1.0	9.0	0	3.0	0
	Cooperative	30.0	27.0	26.0	30.0	30.0	0	0	2.0	4.0	0
**Flora**											
	Unoccupied	0	0	0	0	0	3.0	0	4.0	0	0
	Solitary	2.0	3.0	12.0	6.0	10.0	0	10.0	16.0	21.0	15.0
	Onlooker	0	2.0	4.0	1.0	0	5.0	10.0	4.0	3.0	0
	Parallel	0	5.0	8.0	3.0	6.0	22.0	10.0	10.0	6.0	10.0
	Associative	26.0	18.0	5.0	16.0	21.0	0	0	0	1.0	5.0
	Cooperative	2.0	2.0	1.0	4.0	3.0	0	0	0	0	0
**Beau**											
	Unoccupied	0	0	0	0	0	0	0	0	0	0
	Solitary	0	0	0	0	0	23.0	19.0	16.0	13.0	25.0
	Onlooker	0	0	0	0	0	7.0	2.0	2.0	1.0	0
	Parallel	0	2.0	4.0	0	0	0	5.0	7.0	10.0	0
	Associative	17.0	13.0	17.0	11.0	6.0	0	4.0	5.0	6.0	0
	Cooperative	13.0	15.0	9.0	19.0	24.0	0	0	0	0	5.0

The data for the community playground show a clearly differentiated pattern. Specifically, all six participants were observed playing alone for the majority of the time on the community playground (Deiondre—65%, Julia—63%, Tamisha—68%, Tobias—65%, Flora—41%, Beau—64%). Julia, Tamisha, and Flora spent some time in parallel play. This occurred when the girls were observed on the swings. Deiondre, Tobias, and Beau were more likely to watch other children use the zipline (onlooker play) or jumped off or rolled down the turf hill after observing peers jump or roll down the hill (parallel play). Brief moments of associative play on the community playground occurred when study participants briefly agreed to chase a peer around the playground. The chasing was not consistent, and the study participants often lost track of who they were chasing.

### Interview Data

The majority of codes were categorized under “*peer interactions,” “playground equipment,”* and *“descriptions of future playgrounds”* along with subcategories. Themes and subcategories that were not associated with the interview schedule, but rather were spontaneously derived through the discussions with the children, are noted in the presentation of findings. This section presents data from the following main themes: (1) *interactions with peers*, *(*2) *perspectives on playground equipment*, and (3) *descriptions of future playgrounds*.

#### Interactions With Peers

This theme was derived from elicited and spontaneous discussions of who the children interacted with on the playground. Responses were organized into three subcategories: (1) interactions with friends, (2) interactions with adults, and (3) strangers.

#### Interactions With Friends

All six study participants recognized and described features associated with having friends to play with on the playground. For instance, when asked, *“who do you play with on the playground?,”* all children mentioned names of peers they played with on the school playground and activities they did while playing with these peers (e.g., basketball, tag, gaga pit ball, play structure). Despite being interviewed on the community playground, none of the children mentioned names of peers they played with on the community playground. They also did not mention any activities they did with peers on the community playground. When asked directly if they played with any children while on the community playground, none of the children said they played with peers. The children, as a whole, were more talkative about their friends at school and what they did with these friends on the school playground.

#### Interactions With Adults

Children shared that they interacted more frequently with their parents on the community playground than teachers on the school playground. For some children, the parent interactions were a means to demonstrate their abilities on the community playground (e.g., “*I told my mom I goed fast down the slide;” “Watch me do it.”)*. For others, adult interactions were a means to get their parent to play with them on the community playground (e.g., “*I raced my dad on the track*”). The two children (Beau, Tobias) who mentioned an adult interacting with them on the school playground indicated that the teacher was resolving a conflict with a peer (e.g., “*Who had the ball first?”*).

#### Strangers

All six children highlighted that they did not know any of the children at the community playground. For example, when prompted to discuss, “*who they played with at* *name of community playground*,” four children spontaneously commented about how they were not permitted to play with strangers (e.g., “*I do not know them. I’m not allowed to talk to strangers;” “They’re strangers”)*. The children also mentioned how they do not prefer to play with children they do not know (e.g., “*Becca* [name of friend] *is not here. I only play with Becca. Not kids I do not know;” “I do not know them. I do not want to play with them”*). The children did not mention strangers when discussing the school playground.

### Perspectives on the Playground

To ascertain the children’s perspectives on preferred and not preferred playground activities, children were asked what they liked most and least about each playground. All participants were prompted by the interviewer to discuss their most and least liked playground activities with the prompts, “*What do you like to play on at the school and name of community playground?*” and “*What do you not like to play on at the school and name of community playground?*” All children expressed that they liked the zipline the most at the community playground. The most liked activity at the school playground varied by gender. The boy participants liked basketball (Deiondre) or the gaga ball pit (Tobias, Beau). One girl (Flora) liked the playground structure best. Julia said, “*I like walking around with Becca.”* This statement does not specify any activity or piece of playground equipment but reiterates her friendship. Tamisha answered this question by saying, “*I like playing house,”* which indicates she is participating in social dramatic play while on the school playground.

Answers to their least liked activity at the school and community playground varied. For the school playground, Beau and Tobias selected the playground structure, Deiondre selected the gaga ball pit, and Tamisha selected the four-square activity (painted four-square on the asphalt). Julia and Flora both answered basketball with Julia adding, “*The boys never let the girls play basketball. I do not like it.”* For the community playground, Beau and Tobias selected the swings, Deiondre selected the infant/toddler area, Tamisha and Flora selected the musical instruments, and Julia selected the disk swing.

### Description of Future Playgrounds

This theme encompassed the ideas the children had for future playgrounds. Children were asked, “*what changes would you make to your school’s playground?”* and “*what changes would you make to the* *name of community playground*?” This theme was divided into two subcategories: (1) play and (2) friendship.

#### Play

A similar pattern of responses emerged with regard to children’s descriptions of future playgrounds. Most children expressed a desire to play on a “*fun playground,”* but when asked to define a fun playground, responses basically centered on moving the community playground to the school playground’s location. For example, one child answered, “*I just want name of community playground to move to my school.”* A different child responded, “*Can I move my school here?”* She wanted to move her elementary school to the community playground site.

When children were prompted with, “*Since we cannot move name of the community playground to your school, what playground pieces from name of the community playground would you like to have at your school?”* the children said the zipline, the large playground structure, the turf hill, and the sensory maze. The children were then asked if they would keep any of the playground equipment that is currently at their school. None of the children indicated that they would keep any of their school’s current playground equipment.

#### Friendship

The children repeatedly asked if they could bring their school friends to the community playground. For instance, Julia said, “*Can I bring Becca here*?” Other children asked if they could take a school trip here (meaning the community playground) so they could play with their friends. Overall, these findings suggest that friends are as equally important to preschoolers as the playground equipment on a playground setting.

## Discussion

The aims of the current study were to develop an in-depth understanding of: (1) children’s social play behaviors on school and community playgrounds, (2) the duration with which children play within varying social play categories, and (3) assessing children’s perspectives of playground activities, their peer relationships, and recommendations for new playgrounds. Specifically, the study explored how children played on a school playground and whether this play behavior was the same or different from their play on a community playground. This study was guided by a theoretical framework of affordances that acknowledges the need to discover the range of children’s play abilities under different contextual conditions (Bretherton et al., 1984). As previously noted, setting influences how play is expressed and play in one setting may be more indicative of children’s play competence than play in another setting (Malone and Stoneman, 1990; Malone, 2006).

The direct observation results support and extend previous work, indicating that children’s play skill competence varies by setting (Wilson, 2015). Children in the current study demonstrated higher levels of associative and cooperative play on the school playground, but higher levels of solitary and parallel play on the community playground. This difference in play styles by playground appears to be a function of available play partners and is explained by the interview data. All six participants identified who they played with on the school playground but indicated they did not play with any child on the community playground. Peer familiarity is a contextual variable that has received limited attention in the literature (since the early 80s) despite being a variable that influences play outcomes (Doyle et al., 1980). More specifically, peer familiarity is a noteworthy variable as higher-level play abilities (e.g., associative play, cooperative play) appear to covary with one another. Indeed, with a familiar peer, children tend to have: (1) higher levels of social interactions, (2) more complex cognitive levels of play, (3) increased positive peer interactions, and (4) more successful peer relationships (Doyle et al., 1980).

Our duration findings suggest that play with friends or familiar peers leads to longer episodes of higher-quality play (e.g., cooperative, associative). While this result is not unexpected since the definitions for associative and cooperative play require peer interaction, it does provide an initial area for further study—the amount of time and the level of engagement that is needed for children to feel comfortable enough to play with strangers. In the interview data, participants frequently mentioned the strangers that they experienced at the community playground. Some participants indicated that they could not play with the children at the community playground because they are not permitted to interact with the strangers, whereas other children said they are not comfortable playing with children they do not know. The children repeatedly asked if they could bring their school friends to the community playground or transfer the community playground to their school’s location. These findings signal support for the notion that children enjoyed their play time at the community playground but would prefer it more if they visited this playground with friends or familiar peers. However, given that many participants were observed in parallel and/or onlooker play at the community playground, it is possible that parallel and onlooker play serve as a different form of peer involvement on a community playground (Harper and Huie, 1985). It is possible that preschoolers use parallel and/or onlooker play as a means of getting more comfortable with unfamiliar children with the hope of receiving an initiating play bid from the peer. As previously mentioned, these findings call for a line of research aimed at examining the amount of time and the level of engagement that is needed for children to feel comfortable enough to play with unfamiliar peers. Focusing research efforts in this direction has potential to meaningfully inform peer relationship pedagogy on playground environments and support children’s overall play skill abilities. The knowledge from these study findings suggests to playground manufacturers that associative and cooperative play does not “magically” happen due to the presence of playground equipment, but rather to the peer relationships that children have with others on the playground setting. While play materials may influence the general form of play such as pretend play or constructive play, it is the familiarity of the playmate that influences the overall social quality of the play (Veiga et al., 2017).

### Limitations and Future Studies

The present study had several limitations. In particular, the small sample (*N*=6) made it impossible to state definitively the ways in which student demographics and play abilities could be related to specific results of the study. Our observations occurred at only one school playground and one community playground in a large midwestern city. The two playgrounds have notable qualitative differences between the two play settings. Observations across a greater number of playgrounds would have provided information across more varied environments. The community playground was located within 15min (driving distance) from the school playground. It is possible that a closer community playground would have yielded familiar peers to the participants. Future studies should be conducted with varying types of community playgrounds (e.g., neighborhood, destination) to determine whether similar or different results are found.

### Implications for Researchers, Policymakers, and Urban Planners

Although the current study only investigated the social play behaviors of six participants in a single school playground and community playground context, the results do provide some important observational and interview data regarding preschool children. It is one of the first studies in recent years to gather systematic data from preschool children in two different playground contexts—a school playground and a community playground, at one time. All six participants had highly sophisticated social play behavior skills. They were able to play cooperatively with peers on the school playground. Interview data indicated that they preferred the playground activities at the community playground more, but their social play behavior was in the solitary play range with some examples of onlooker and parallel play while visiting the community playground. For researchers, this study provides a foundation for additional studies about the correlation between contextual factors (e.g., playground type) and social play behavior differences in preschoolers. Specifically, future studies examining cognitive and/or social play on playgrounds must consider how contextual factors such as peer familiarity may influence the study’s results.

For policymakers and urban planners, this study helps identify playground equipment preferences for school and community playgrounds. Interview data indicated that the children preferred the playground activities available at the community playground. Although adults select and purchase the playground equipment when building new playgrounds, the consumers of the playground equipment on these environments are children. Child input into product selection may lead to better child outcomes in a variety of areas. This is an area of research that may be worth exploring in the future.

## Data Availability Statement

The datasets presented in this article are not readily available because The University of Cincinnati does not allow transcribed and coded qualitative data to be shared due to confidentiality purposes. Requests to access the datasets should be directed to \tina.stanton-chapman@uc.edu.

## Ethics Statement

The studies involving human participants were reviewed and approved by IRB, University of Cincinnati. Written informed consent to participate in this study was provided by the participants’ legal guardian/next of kin.

## Author Contributions

TS-C contributed to the conception and design of the study. ES organized all incoming data. TS-C wrote the first draft of the manuscript except the setting. ES wrote the setting descriptions in the first draft of the manuscript. TS-C and ES contributed to manuscript revision, read, and approved the submitted version.

## Conflict of Interest

ES was employed by the company Playground Equipment Services Inc.

The remaining author declares that the research was conducted in the absence of any commercial or financial relationships that could be construed as a potential conflict of interest.

## Publisher’s Note

All claims expressed in this article are solely those of the authors and do not necessarily represent those of their affiliated organizations, or those of the publisher, the editors and the reviewers. Any product that may be evaluated in this article, or claim that may be made by its manufacturer, is not guaranteed or endorsed by the publisher.

## References

[ref1] Ayres K. Gast D. L. (2010). “Dependent measures and measurement procedures,” in Single Subject Methodology in Behavioral Sciences. ed. Gast D. L. (New York: Routledge), 129–165.

[ref2] Barone D. (2004). “Case study research,” in Literacy Research Methodologies. eds. Duke N. Mallette M. (New York: Fuilford Press), 7–27.

[ref3] Bourke T. M. Sargisson R. J. (2014). A behavioral investigation of preference in a newly designed New Zealand playground. Am. J. Play 6, 370–391.

[ref4] Bretherton L. O'Connell B. Shore C. Bates E. (1984). “The effect of contextual variation on symbolic play: development from 20 to 28 months,” in Symbolic Play: The Development of Social Understanding. ed. Bretherton I. (New York: Academic Press), 271–298.

[ref5] Brown J. G. Burger C. (1984). Playground designs and preschool children’s behavior. Environ. Behav. 16, 599–626.

[ref7] Cohen J. (1960). A coefficient of agreement for nominal scales. Edu. Psychol. Meas. 20, 37–46.

[ref8] Creswell J. W. (2009). Research Design: Qualitative, Quantitative and Mixed Methods Approaches. Los Angeles: Sage Publications.

[ref9] Creswell J. W. Clark V. P. (2017). Designing and Conducting Mixed Methods Research. Los Angeles: Sage Publications.

[ref10] Czalczynska-Podolska M. (2014). The impact of playground spatial features on children’s play and activity forms: An evaluation of contemporary playgrounds’ play and social value. J. Environ. Psychol. 38, 132–142. doi: 10.1016/j.jenvp.2014.01.006

[ref11] Doyle A. Connolly J. Rivest L. (1980). The effect of playmate familiarity on the social interactions of young children. Child Dev. 51, 217–223. doi: 10.2307/1129609

[ref12] Dyson A. H. (1985). “Children out of bounds: The power of case studies in expanding visions of literacy development,” in Perspectives on Literacy Research and Practice. eds. Hinchman K. Leu D. Kinzer C. (Chicago, IL: National Reading Conference), 39–53.

[ref13] Eberle S. G. (2014). The elements of play: Toward a philosophy and a definition of play. J. Play 6, 214–233.

[ref14] Eder D. Corsaro W. (1999). Ethnographic studies of children and youth: theoretical and ethical issues. J. Contemp. Ethnogr. 28, 520–531.

[ref15] Farley T. A. Meriwether R. A. Baker E. T. Rice J. C. Webber L. S. (2008). Where do the children play? The influence of playground equipment on physical activity of children in free play. J. Phys. Act. Health 5, 319–331. doi: 10.1123/jpah.5.2.319, PMID: 18382040

[ref16] Fewell R. R. Ogura T. Notari-Syverson A. Wheeden C. A. (1997). The relationship between play and communication skills in young children with Down syndrome. Top. Early Child. Spec. Educ. 17, 103–118. doi: 10.1177/2F027112149701700109

[ref17] Fromberg D. Bergen D. (eds.). (2006). Play from Birth to Twelve: Contexts, Perspectives, and Meanings. 2nd Edn. New York: Garland Press.

[ref18] Gibson J. J. (1950). The Perception of the Visual World. New York: Houghton Mifflin.

[ref19] Graham M. Wright M. Azevedo L. B. Macpherson T. Jones D. Innerd A. (2021). The school playground environment as a driver of primary school children’s physical activity behaviour: A direct observation case study. J. Sports Sci. 3, 1–13. doi: 10.1080/02640414.2021.1928428, PMID: 34080956

[ref20] Guralnick M. J. Groom J. M. (1988). Peer interactions in mainstreamed and specialized classrooms: A comparative analysis. Except. Child. 54, 415–425. 3342826 10.1177/001440298805400504

[ref21] Harper L. V. Huie K. S. (1985). The effects of prior group experience, age, and familiarity on the quality and organization of preschoolers’ social relationships. Child Dev. 56, 704–717. doi: 10.2307/1129760

[ref22] Holst J. (2017). The dynamics of play: Back to the basics of playing. Int. J. Play 6, 85–95. doi: 10.1080/21594937.2017.1288383

[ref23] Holt N. L. Lee H. Mollar C. A. Spence J. C. (2015). ‘Eyes on where children play:’ A retrospective study of active free play. Children’s Geo. 13, 73–88. doi: 10.1080/14733285.2013.828449

[ref24] Jarusriboonchai P. Meissner J. L. Almeida T. Balaam M. (2019). Understanding children’s play in primary schools, in *CandT: Proceedings of the 9th International Conference on Communities and Technologies*; June 17, 2019.

[ref25] Johnson J. E. Christie J. F. Yawkey T. D. (1999). Play and Early Childhood Development. 2nd *Edn*. New York: Allyn and Bacon.

[ref26] Kelly S. E. (2013). “Qualitative interviewing techniques and styles,” in The Sage Handbook of Qualitative Methods in Health Research. eds. Bourgeault I. Dingwall R. Vries R. de (London: Sage Publications), 307–326.

[ref27] Lambert V. Glacken M. McCarron M. (2013). Meeting the information needs of children in hospital. J. Child Health Care 17, 338–353. doi: 10.1177/2F1367493512462155 23411658

[ref28] Lasenby-Lessard J. Morrongiello B. (2011). Understanding risk compensation in children: experience with the activity and level of sensation seeking play a role. Accident Anal. Prev. 43, 1341–1370. doi: 10.1016/j.aap.2011.02.006 21545863

[ref29] Lasenby-Lessard J. Morrongiello B. A. Barrie D. (2013). The impact of accumulated experience on children's appraisals of risk and risk-taking decisions: implications for youth injury prevention. Health Psychol. 32, 370–378. doi: 10.1037/a0023857 21604879

[ref30] Malone D. M. (1999). Contextual factors informing play-based program planning. Int. J. Disabil. Dev. Educ. 46, 307–324.

[ref31] Malone D. M. (2006). Contextually influenced patterns of play developmental age associations for preschoolers with and without mental retardation. Early Childhood Educ. J. 34, 215–225. doi: 10.1007/s10643-006-0134-7

[ref01] Malone D. M. Langone J. (1998). Variability in the play of preschoolers with cognitive delays across different toy sets. Int. J. Disabil. Dev. Educ. 45, 127–142.

[ref32] Malone D. M. Stoneman Z. (1990). Cognitive play of mentally retarded preschool children: observations in the home and school. Am. J. Ment. Retard. 94, 475–487. 1690555

[ref33] Malone D. M. Stoneman Z. Langone J. (1994). Contextual variation of correspondences among measures of play and developmental level of preschool children. J. Early Interv. 18, 199–215.

[ref34] Palinkas L. A. Horwitz S. M. Green C. A. Wisdom J. P. Duan N. Hoagwood K. (2016). Purposeful sampling for qualitative data collection and analysis in mixed method implementation research. Adm. Policy Ment. Health Ment. Health Serv. Res. 42, 533–544. doi: 10.1007/s10488-013-0528-y, PMID: 24193818 PMC4012002

[ref35] Parten M. (1932). Social participation among preschool children. J. Abnorm. Soc. Psychol. 28, 136–147.

[ref36] QSR International. (2008). InVivo 8. Available at: https://www.qsrinternational.com/nvivo-qualitative-data-analysis-software/home?_ga=2.156632255.540643659.1627186796-2142655888.1627186796 (Accessed July 18, 2021).

[ref37] Ridgers N. D. Stratton G. Fairclough S. J. (2006). Physical activity levels of children during school playtime. Sports Med. 36, 359–371. doi: 10.2165/00007256-200636040-00005, PMID: 16573359

[ref38] Rimmer J. (2005). Exercise and physical activity in persons aging with a physical disability. Phy. Med. Rehab. Clin. North America 16, 41–56. doi: 10.1016/j.pmr.2004.06.013, PMID: 15561544

[ref39] Rubin K. Fein G. Vandenberg B. (1983). “Play,” in Handbook of Child Psychology: Socialization, Personality, Social Development. *Vol*. 4. ed. Hetherington E. M. (New York: Wiley), 694–759.

[ref41] Sallis J. F. Conway T. L. Prochska J. J. McKenzie T. L. Marshall S. J. Brown M. (2001). The association of school environments with youth physical activity. Am. J. Public Health 91, 618–620. doi: 10.2105/ajph.91.4.618, PMID: 11291375 PMC1446652

[ref42] Sando O. J. (2019). The physical indoor environment in ECEC settings: Children’s well-being and physical activity. Eur. Early Childhood J. 27, 506–519. doi: 10.1080/1350293X.2019.1634238

[ref43] Sandseter E. B. H. (2009). Affordances for risky play in preschool: The importance of features in the play environment. Early Childhood Educ. J. 36, 439–446. doi: 10.1007/s10643-009-0307-2

[ref44] Stanton-Chapman T. L. Schmidt E. L. (2016). Special education professionals’ attitudes towards accessible playgrounds and recreational facilities. Res. Prac. Persons Severe Dis. 41, 90–100. doi: 10.1177/2F1540796916638499

[ref45] Stanton-Chapman T. L. Schmidt E. L. (2017). Caregiver perceptions of inclusive playgrounds targeting toddlers and Preschoolers with disabilities: has recent international and National Policy Improved Overall Satisfaction? J. Res. Spec. Educ. Needs 17, 237–246. doi: 10.1111/1471-3802.12381

[ref46] Stone S. J. (2017). The essential role of play in school contexts for the well-being of children. Learn. Landscapes 10, 315–318. doi: 10.36510/learnland.v10i2.817

[ref47] Uys M. Draper C. E. Hendricks S. de Villiers A. Fourie J. Steyn N.. (2015). Factors influencing break-time physical activity of south African primary school learners from low-income communities. J. Phys. Act. Health 12, 618–627. doi: 10.1123/jpah.2013-0188, PMID: 24904983

[ref48] Veiga G. de Lang W. Cachucho R. Ketelaar L. Kok J. N. Knobbe A.. (2017). Social competence at the playground: Preschoolers at recess. Infant Child Dev. 26:e1957. doi: 10.1002/icd.1957

[ref49] Wilson H. E. (2015). Patterns of play behaviors and learning center choices between high ability and typical children. J. Adv. Acad. 26, 143–164. doi: 10.1177/2F1932202X15577954

[ref50] Yuill N. Strieth S. Roake C. Aspden R. Todd B. (2007). Brief report: designing a playground for children with autistic spectrum disorders- effects on playful peer interactions. J. Autism Dev. Disorder 37, 1192–1196. doi: 10.1007/s10803-006-0241-8, PMID: 17063401

